# Measles incidence and measles-containing vaccine coverage in Chereponi District, Ghana, 2019-2024: A retrospective analysis of routine surveillance and immunization data

**DOI:** 10.1016/j.gloepi.2026.100286

**Published:** 2026-08-22

**Authors:** Richard Dzeha, Owusu Amponsah

**Affiliations:** a Regional Health Information Unit, North East Regional Health Directorate, Gambaga, Ghana; b Department of Planning, Kwame Nkrumah University of Science and Technology, Kumasi, Ghana

**Keywords:** Measles, Measles-containing vaccine, Vaccination coverage, Disease surveillance, Measles incidence, Ghana

## Abstract

**Objectives:**

To describe temporal patterns in measles occurrence and measles-containing vaccine (MCV) coverage in Chereponi District, Ghana, from 2019 to 2024.

**Methods:**

A retrospective descriptive study analysed routine SORMAS and DHIMS2 data from 2019 to 2024. Annual suspected and laboratory-confirmed measles incidence, MCV1 and MCV2 coverage, and MCV1-MCV2 dropout were summarized descriptively.

**Results:**

During 2019–2024, 167 suspected measles cases were reported through district surveillance, of which 31 (18.6%) were laboratory-confirmed. Children aged 0–4 years accounted for 66.9% of suspected cases with recorded age, while 5–9-year-olds accounted for 23.4%. Suspected notifications peaked in 2022, whereas laboratory-confirmed incidence was highest in 2023. MCV1 reached 95% only in 2020, while MCV2 remained below 90% in four of six years. MCV1-MCV2 dropout exceeded 10% in four years and declined to 8.6% in 2024.

**Conclusion:**

Chereponi experienced substantial year-to-year variation in measles occurrence amid inconsistent two-dose MCV coverage and periods of high dropout. Strengthening routine immunization, defaulter tracing, outreach, denominator validation, and case-based surveillance remains essential for measles elimination.

## List of abbreviations

**Table:** 

CDC	Centres for Disease Control and Prevention
CHPS	Community-Based Health Planning and Services
DHIMS2	District Health Information Management System II
GHS	Ghana Health Service
IDSR	Integrated Disease Surveillance and Response
MCV	Measles-Containing Vaccine
MCV1	Measles-Containing Vaccine First Dose
MCV2	Measles-Containing Vaccine Second Dose
PPV	Positive Predictive Value
SORMAS	Surveillance Outbreak Response Management and Analysis System
UNICEF	United Nations Children's Fund
WHO	World Health Organization

## Introduction

Measles remains one of the most contagious viral diseases globally and continues to pose a significant public health threat despite the availability of a safe and effective vaccine [1]. The disease is transmitted through respiratory droplets and can remain infectious in the air for up to two hours, contributing to its rapid spread in susceptible populations [2]. An infected individual can transmit the virus to between 12 and 18 other persons, highlighting its high transmissibility and epidemic potential [3]. Globally, measles continues to cause substantial morbidity and mortality, particularly among children under five years of age, despite considerable progress toward elimination [4].

Vaccination with measles-containing vaccines (MCVs), including the first (MCV1) and second (MCV2) doses, remains the most effective strategy for preventing measles infection and achieving herd immunity. Sustaining high vaccination coverage is essential to interrupt transmission; however, declining coverage and immunity gaps have contributed to the resurgence of measles in several settings [5], [6]. Inadequate immunization coverage, vaccine hesitancy, and inequities in access to healthcare services continue to undermine global and national measles elimination efforts [7], [8].

Public health surveillance systems are central to measles control and elimination. Surveillance enables the continuous and systematic collection, analysis, and interpretation of health data for timely public health action [4]. In the context of measles, surveillance systems facilitate early detection of suspected cases, identification of outbreak patterns, and monitoring of vaccination coverage [3], [5]. These functions are essential for guiding targeted interventions, optimizing resource allocation, and evaluating the effectiveness of immunization programs [9], [10]. Robust surveillance systems also support outbreak preparedness and response, thereby minimising disease transmission and associated health system burdens [11].

In Ghana, measles surveillance is implemented within the Integrated Disease Surveillance and Response (IDSR) framework and supported by DHIMS2 and SORMAS [12]. National experience illustrates both progress and continuing vulnerability. Published Ghanaian data reported confirmed measles incidence of 2.7 per 1,000,000 population in 2020, 38.8 per 1,000,000 in 2021 and 11.8 per 1,000,000 in 2022 [13].

Ghana conducted a nationwide measles-rubella campaign in October 2018 targeting more than four million children aged 9 months to under 5 years [14]. A further nationwide integrated measles-rubella and vitamin A campaign was conducted from 2 to 6 October 2024 for children aged 9–59 months; WHO subsequently reported 97.1% administrative campaign coverage nationally, while a post-campaign survey estimated 84% coverage [15]. District-specific SIA coverage for Chereponi was not available in the analytical dataset used for this study.

Chereponi District in the North East Region of Ghana is a rural border district where geographic access, cross-border service use and population movement may affect both immunization delivery and administrative denominators. Evidence from the neighbouring Savannah Region also shows that incomplete measles-rubella vaccination can reflect caregiver knowledge, distance to services, health-worker interactions and vaccination-session disruptions [13]. These contextual factors are relevant when interpreting routine MCV coverage but were not measured directly in the present descriptive analysis. District-level evidence from rural border populations is rarely represented in national summaries; documenting local heterogeneity can identify operational gaps that national averages conceal and generate programme-relevant questions for subsequent analytical studies.

Although district-level routine data are valuable for programme monitoring, interpretation of annual administrative vaccination coverage alongside measles occurrence is complicated by denominator uncertainty, supplementary campaigns, surveillance performance and the aggregated nature of routine data. A closely comparable analysis from the neighbouring Savannah Region described declining routine measles-rubella coverage and increasing dropout before a 2022 outbreak [16]. Building on this evidence, the present study focuses on describing Chereponi's temporal measles patterns and district- and subdistrict-level immunization performance.

This study therefore aimed to characterize temporal patterns in suspected and laboratory-confirmed measles occurrence alongside routine MCV1 and MCV2 coverage and MCV1-MCV2 dropout in Chereponi District from 2019 to 2024. The 2019–2024 period was selected to provide a complete six-year series following establishment of the North East Region, while spanning the COVID-19 disruption and the 2022–2023 resurgence in measles notifications. As a descriptive study, it did not test a causal hypothesis. Its contribution is the integration of six years of district surveillance with district- and subdistrict-level immunization indicators in a mobile rural border setting, while transparently demonstrating how denominator uncertainty, implausible administrative coverage and incomplete case-classification fields affect local measles-elimination monitoring.

## Methods

### Study design

This retrospective descriptive study analysed routinely collected measles surveillance and immunization data for Chereponi District from January 2019 through December 2024. The primary purpose was to characterize temporal epidemiological and immunization programme patterns; no causal or associational hypothesis was tested.

### Study setting

The study was conducted in Chereponi District, North East Region, Ghana, a predominantly rural district bordering the Republic of Togo. In 2024, the district population was projected to be about 96,000 people, and district administrative records described more than 200 communities. The population figure is a projection rather than a census count and is therefore reported in rounded form; its uncertainty reflects assumptions about fertility, mortality and migration since the census. The community description is also approximate because settlements may be created, merged, renamed or differently classified across administrative registers, and an exact contemporaneous count was unavailable. Healthcare services are delivered through a district hospital, health centres, and CHPS compounds operating under Ghana's Integrated Disease Surveillance and Response (IDSR) system.

### Data sources and collection

The study used secondary data extracted from Ghana's routine health information systems. Measles surveillance data, including suspected and laboratory-confirmed cases, were obtained from the Surveillance Outbreak Response Management and Analysis System (SORMAS), while immunization data for the first and second doses of measles-containing vaccine (MCV1 and MCV2) were retrieved from the District Health Information Management System II (DHIMS2). Annual population estimates were obtained from the Ghana Statistical Service population projections.

Data covered January 2019–December 2024 and consisted of aggregated district-level records on suspected measles cases, laboratory-confirmed cases, MCV1 and MCV2 coverage, and population estimates. No sampling was undertaken for the surveillance component; the analysis included all suspected measles cases recorded in SORMAS for Chereponi District during the study period that were available in the extracted analytical dataset. Case-based variables available for descriptive analysis included age and sex. Vaccination status of individual suspected cases, epidemiological linkage, clinically compatible classification, specimen adequacy, investigation within 48 h, and non-measles febrile rash illness counts were not available. Consequently, standard WHO indicators requiring those fields could not be calculated. Before analysis, annual aggregates were checked for missing years, duplicate summary rows, arithmetic consistency between counts and percentages, plausible ranges, and agreement between values reported in the text, tables and figure. All 167 suspected-case records had sex and laboratory-result fields, while age was available for 154 (92.2%). Coverage values above 100% and negative dropout values were retained and flagged rather than recoded because they may reflect denominator error, cross-border service use, migration, reporting delays or non-equivalent dose cohorts. No independent source was available to validate individual records, dose numerators or annual target-population denominators.

### Study variables

The primary surveillance outcomes were annual suspected measles notification incidence and laboratory-confirmed measles incidence, each expressed per 1,000,000 population. Suspected notifications were retained as a surveillance workload/sensitivity measure and were not interpreted as measles disease incidence. Laboratory-confirmed incidence included only cases with laboratory confirmation; because epidemiologically linked and clinically compatible classifications were unavailable, it should not be interpreted as total confirmed measles incidence under the full WHO case-classification framework. Vaccination coverage was the administratively reported proportion of eligible children receiving MCV1 or MCV2. Coverage was summarized for Bumburiga, Chereponi, Garinkuka, Wenchiki and Wonjuga subdistricts and for the district overall.

Immunization programme indicators included MCV1-MCV2 dropout, calculated as [(MCV1 doses − MCV2 doses)/MCV1 doses] × 100. Negative values therefore occur when reported MCV2 doses exceed MCV1 doses and were retained as signals of cohort mismatch, migration, reporting timing or other data-quality limitations rather than interpreted as true negative dropout. The proportion of suspected cases with a laboratory result was also described. Specimen adequacy, investigation within 48 h and the non-measles febrile rash illness rate could not be calculated because the required variables were not present in the extracted dataset. Annual population estimates were used as incidence denominators. (see Table 1).

**Table 1:** Annual population estimates used for measles incidence calculations, Chereponi District, 2019–2024.

Variable	2019	2020	2021	2022	2023	2024
Population estimate	67,346	68,904	87,176	89,007	90,876	96,218

### Data analysis

Data were cleaned, validated and analysed using Microsoft Excel and Stata version 17. Frequencies, percentages, annual incidence rates and administrative vaccination coverage were summarized in tables and figures. Annual incidence was calculated as the number of reported cases divided by the corresponding annual projected population, multiplied by 1,000,000. Temporal changes were described graphically and descriptively. Given only six annual observations and 31 laboratory-confirmed cases, no formal inferential trend or association analysis was performed. Administrative coverage values above 100% were retained and explicitly interpreted as potential numerator-denominator or reporting problems.

### Ethical considerations

Ethical approval was obtained from the Committee on Human Research, Publication and Ethics, Kwame Nkrumah University of Science and Technology (CHRPE/AP/640/25). The North East Regional and Chereponi District Health Directorates granted administrative permission.

## Results

### Measles incidence trends, 2019–2024

**Fig. 1:**
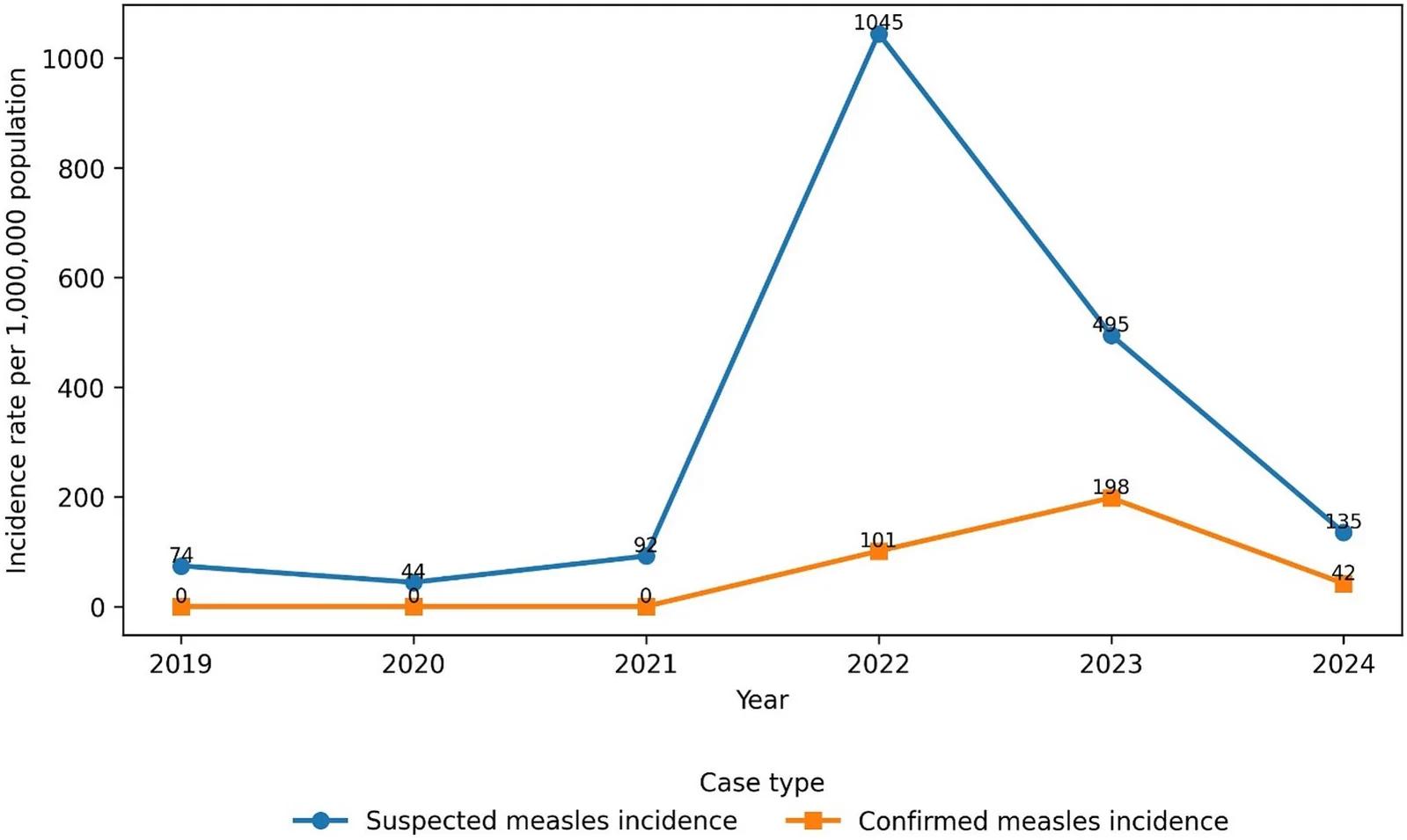
Trends in suspected and laboratory-confirmed measles incidence rates per 1,000,000 population in Chereponi District, Ghana, 2019–2024.

During 2019–2024, district surveillance recorded 167 suspected measles cases, of which 31 (18.6%) were laboratory-confirmed (Table 2; Fig. 1). This percentage is reported as the proportion of suspected records with a positive laboratory result, not as positive predictive value (PPV), because the available data did not support a formal assessment of surveillance predictive performance. Males accounted for 86/167 (51.5%) and females for 81/167 (48.5%). Children aged 0–4 years accounted for 103/154 (66.9%) of records with age captured in the summarized dataset, and those aged 5–9 years accounted for 36/154 (23.4%); the remaining age groups each contributed fewer than 4% of cases. Individual vaccination status was unavailable, so the age distribution and aggregate coverage are presented as parallel descriptive findings only; age-specific coverage, vaccination status among cases and an age-coverage relationship could not be analysed.

**Table 2:** Demographic characteristics and laboratory results of suspected measles cases, Chereponi District, 2019–2024.

Variables	Option	Frequency	Percent
Sex	Male	86	51.5
	Female	81	48.5
Age group (years)	0–4	103	61.67
	5–9	36	21.55
	10–14	6	3.59
	15–19	3	1.79
	20–24	1	0.59
	25–29	2	1.19
	30–34	2	1.19
	45–49	1	0.59
	Missing value	13	7.78
Suspected cases over the period	2019	5	2.99
	2020	3	1.8
	2021	8	4.79
	2022	93	55.69
	2023	45	26.95
	2024	13	7.78
Laboratory confirmation	Positive	31	18.6
	Negative	136	81.4
Total		167	100

Suspected notifications varied from 3 cases in 2020 to 93 in 2022. Suspected notification incidence was 74, 44, 92, 1045, 495 and 135 per 1,000,000 population in 2019–2024, respectively. No laboratory-confirmed cases were recorded in 2019–2021; laboratory-confirmed incidence was 101 per 1,000,000 in 2022, 198 per 1,000,000 in 2023 and 42 per 1,000,000 in 2024. Because these rates were based on only 31 confirmed cases overall and single-digit counts in some years, apparently large year-to-year rate changes are statistically unstable and should not be interpreted as precise underlying trends. All 167 suspected cases had a recorded laboratory result (31 positive and 136 negative); however, specimen adequacy could not be assessed from the available variables (Fig. 1).

### Measles-containing vaccine coverage

Administrative vaccination coverage exceeded 100% in several subdistrict-year observations, particularly in 2020. These values were retained as reported because no alternative denominator series was available for sensitivity analysis. They were therefore interpreted as administrative programme indicators that may be affected by projected target-population error, migration, cross-border service utilization, delayed reporting or cohort mismatch.

#### MCV1 coverage

**Fig. 2:**
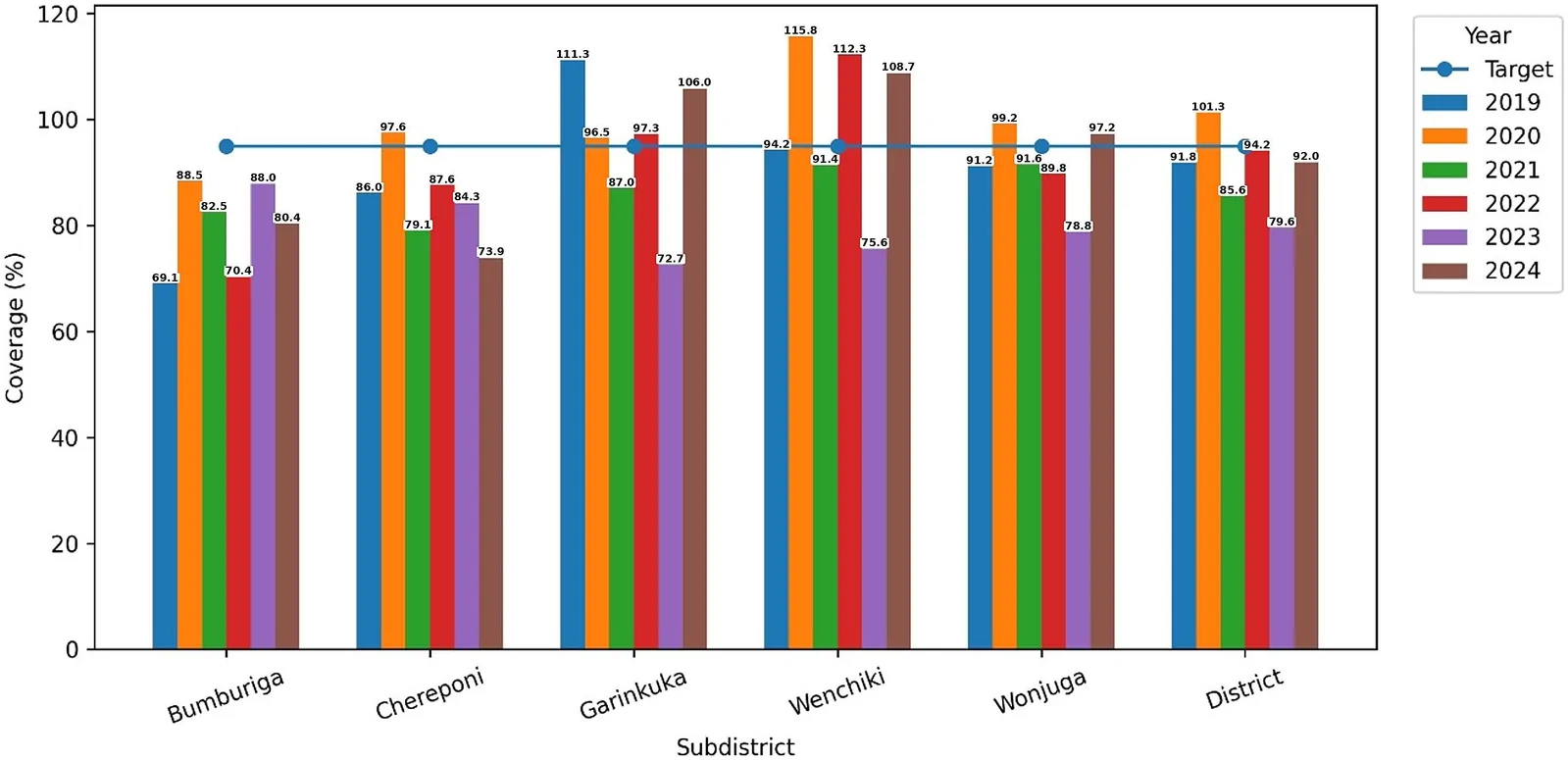
Subdistrict-level trends in measles-containing vaccine first dose (MCV1) coverage in Chereponi District, Ghana, 2019–2024.

District MCV1 coverage fluctuated considerably and did not consistently achieve the WHO target of ≥95% (Fig. 2). Coverage increased from 91.8% in 2019 to 101.3% in 2020 before declining to 85.6% in 2021. It partially recovered to 94.2% in 2022, decreased further to 79.6% in 2023, and improved to 92.0% in 2024, remaining below the recommended threshold.

Subdistrict performance was heterogeneous. Chereponi declined from 97.6% in 2020 to 73.9% in 2024, while Bumburiga consistently remained below target (69.1–88.5%). Garinkuka exceeded 100% in several years but dropped to 72.7% in 2023. Wenchiki consistently achieved high coverage except for a decline in 2023 (75.6%), recovering to 108.7% in 2024. Wonjuga maintained relatively stable coverage, exceeding 90% in most years and reaching 97.2% in 2024. Overall, MCV1 performance remained inconsistent across both the district and subdistricts (Fig. 2).

#### MCV2 coverage

**Fig. 3:**
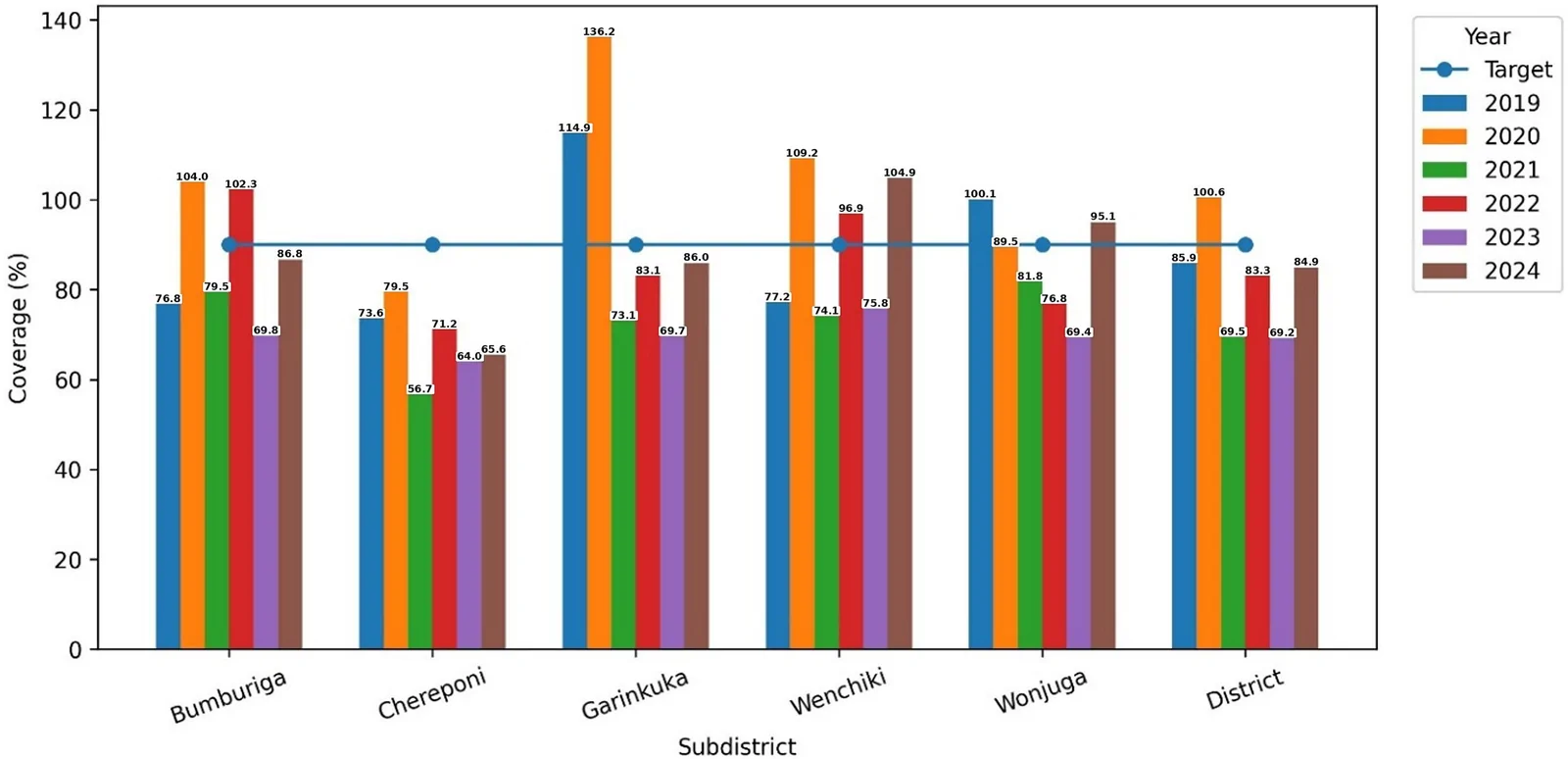
Subdistrict-level trends in measles-containing vaccine second dose (MCV2) coverage in Chereponi District, Ghana, 2019–2024.

MCV2 coverage remained below the WHO target of ≥90% in most years (Fig. 3). District coverage increased from 85.9% in 2019 to 100.6% in 2020 but declined sharply to 69.5% in 2021. Coverage improved slightly to 83.3% in 2022, declined again to 69.2% in 2023, and recovered modestly to 84.9% in 2024.

Marked subdistrict variation was observed. Chereponi subdistrict recorded MCV2 coverage of 56.7%–79.8%, while Bumburiga and Garinkuka showed substantial year-to-year variation, including values above 100% and below 70%. Wenchiki exceeded 90% in several years, including 2020, 2022 and 2024, and Wonjuga reached 97.2% in 2024. These descriptive differences identify areas for programme review but do not establish reasons for the observed coverage patterns (Fig. 3).

### MCV1-MCV2 dropout rates

**Fig. 4:**
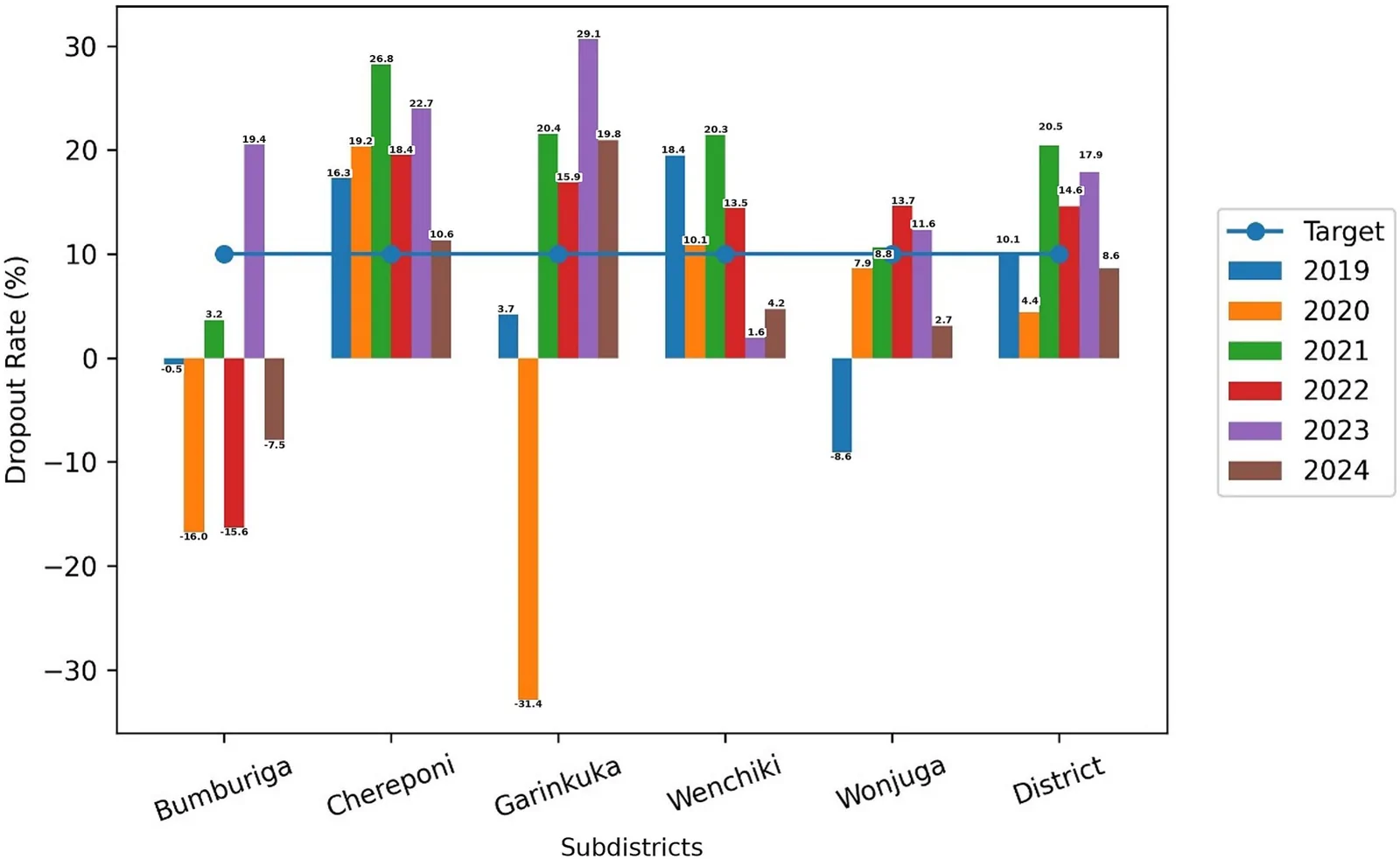
Subdistrict-level trends in measles-containing vaccine dropout rates (MCV1–MCV2) in Chereponi District, Ghana, 2019–2024.

Dropout between MCV1 and MCV2 varied substantially over time and frequently exceeded the WHO recommended threshold of <10% (Fig. 4). District dropout declined from 10.1% in 2019 to 4.4% in 2020 but increased markedly to 20.5% in 2021, remaining elevated in 2022 (14.6%) and 2023 (17.9%). By 2024, dropout declined to 8.6%, meeting the WHO benchmark.

Subdistrict trends revealed persistent disparities. Chereponi consistently recorded high dropout rates (11.3%–28.2%), while Garinkuka also experienced substantial fluctuations (16.9%–30.7%). In contrast, Wenchiki demonstrated sustained improvement, reducing dropout to 1.9% in 2023 and 4.7% in 2024. Wonjuga maintained relatively stable performance, achieving a dropout rate of 3.1% in 2024.

Negative dropout values occurred in Bumburiga and Garinkuka in some subdistrict-year observations, meaning that reported MCV2 doses exceeded MCV1 doses. Under the specified formula, these values are mathematically possible when administrative dose counts do not represent a closed cohort. They were therefore treated as data-quality/programme-monitoring signals rather than evidence of exceptionally high programme retention (Fig. 4).

## Discussion

### Measles incidence

This study identified substantial year-to-year variation in measles notifications and laboratory-confirmed disease in Chereponi District. Notifications rose sharply in 2022, while laboratory-confirmed incidence was highest in 2023. However, only 31 cases were laboratory-confirmed across the six years, and early annual counts were zero or small. The resulting rates are sensitive to the addition or subtraction of a few cases and should be interpreted as descriptive signals rather than evidence of a stable epidemiological trend. The observed patterns may also reflect changes in case detection, reporting, testing, routine immunization, vaccine availability and supplementary vaccination rather than a single mechanism.

The 2020–2021 period coincided with COVID-19-related disruption of routine immunization and surveillance across the WHO African Region [16]. Ghanaian evidence also documents vaccine-supply disruptions and marked national variation in confirmed measles incidence during 2020–2022 [13]. These contextual events may have contributed to immunity and surveillance changes, but the present ecological dataset cannot quantify their independent effects.

A study from Savannah Region, Ghana provides a particularly relevant comparator reported declining routine measles-rubella coverage and increasing MR1-MR2 dropout before a region-wide 2022 measles outbreak [17]. A subsequent caregiver study in Bole and Central Gonja found that only about 56% of sampled children had completed two routine MR doses and identified inadequate vaccination knowledge, distance of at least 5 km to services, poor perceived health-worker attitude and vaccination-session postponement as barriers to completion [13]. These findings provide plausible programme context for northern Ghana, but they should not be assumed to explain Chereponi's patterns because the present study did not measure these determinants.

Age distribution is also important for interpretation. Children aged 0–4 years constituted approximately two-thirds of suspected cases, and those aged 5–9 years a further 23.4%. Routine coverage measured in a given calendar year does not necessarily represent the vaccination exposure of older children who were vaccinated, or should have been vaccinated, in earlier birth cohorts. Because individual vaccination histories and age-specific denominator data were unavailable, the study did not analyse an association between age distribution and coverage. Their juxtaposition is descriptive and should not be interpreted as evidence that annual aggregate coverage explains the age pattern of cases.

The extracted dataset showed a laboratory result for all 167 suspected cases, but it did not contain specimen adequacy, investigation-within-48-h or non-measles febrile rash illness variables. We therefore do not label the laboratory-confirmation proportion as surveillance ‘sensitivity’ or compare it with an 80% sensitivity target. A fuller evaluation of surveillance performance requires standard WHO indicators and complete case-based investigation data.

### Measles immunization coverage

Sustained high population immunity requires both routine two-dose vaccination and supplementary activities. District MCV1 exceeded 95% only in 2020, while MCV2 was below 90% in four of six years. However, these administrative estimates should not be read as direct measures of individual immunity. Ghana conducted nationwide MR SIAs immediately before the study period in October 2018 and again in October 2024 [14], [15]. The 2024 campaign reached children aged 9–59 months and WHO reported 97.1% national administrative coverage, although the post-campaign survey estimate was 84% [15]. Because Chereponi-specific SIA coverage was unavailable, the extent to which these campaigns modified local susceptibility could not be quantified.

Coverage above 100% is an important limitation of administrative data rather than evidence that more than the entire target population was immunized. Such values can arise when dose numerators include children outside projected target populations, while denominator projections fail to capture migration, population growth or cross-border utilization. This issue is especially relevant in a district bordering Togo. The absence of an independent annual denominator series prevented a formal sensitivity analysis, so the reported coverage should be interpreted as programme-monitoring estimates.

MCV2 consistently lagged behind MCV1 at district level, although the size of the gap varied by year and subdistrict. The Savannah and Upper East Regions of Ghana caregiver study provides complementary evidence that completion of the second dose can be affected by caregiver knowledge, geographical access, service reliability and health-worker interactions [13], [18]. These findings support targeted programme assessment in low-performing subdistricts, rather than assuming that coverage gaps arise from a single mechanism.

### MCV1-MCV2 dropout

Dropout between MCV1 and MCV2 is an important indicator of immunization programme performance, with WHO recommending rates below 10% [17]. Dropout in Chereponi District frequently exceeded this threshold, peaking at 20.5% in 2021 before improving to 8.6% in 2024.

District dropout exceeded 10% in 2019, 2021, 2022 and 2023 and declined to 8.6% in 2024. Negative subdistrict dropout values in Bumburiga and Garinkuka occurred because reported MCV2 doses exceeded MCV1 doses. This can occur with administrative data when the two dose counts do not refer to an identical closed cohort because of migration, catch-up vaccination, reporting timing or denominator problems. Programme managers should therefore examine dose-level and cohort data before using negative dropout values to infer performance.

### Study strengths and limitations

This study has several strengths. It integrates six consecutive years of routine measles surveillance and immunization programme data from a defined district population and includes both district- and subdistrict-level immunization indicators. Rather than relying on a sampled surveillance population, the analysis included all reported suspected measles cases available in the district surveillance dataset during the study period. The analysis also explicitly accounts for important limitations of administrative coverage estimates and maintains cautious interpretation of patterns observed in aggregated routine data.

Nevertheless, several limitations and potential sources of bias should be noted. First, passive surveillance may under-ascertain cases when ill persons do not seek care, clinicians do not recognize or report suspected measles, or access barriers affect presentation; temporal changes in case finding and reporting could therefore mimic changes in occurrence. Second, restricting confirmed incidence to laboratory-positive records may misclassify total measles burden because epidemiologically linked and clinically compatible classifications and specimen-adequacy data were unavailable. Third, missing age for 13 suspected cases (7.8%) and absent vaccination histories may bias demographic description and prevented individual-level linkage of vaccination and disease. Fourth, administrative coverage is vulnerable to numerator error, duplicate or delayed reporting, vaccination of children outside the target population, and denominator error from projected populations, migration and cross-border service use; values above 100% and negative dropout demonstrate this limitation. Fifth, annual aggregation creates ecological and temporal mismatch: children counted as cases may not belong to the cohort represented by the same year's coverage. The 2018 and 2024 supplementary campaigns, COVID-19 disruption and other unmeasured programme changes may also confound visual comparisons over time. Sixth, the small number of confirmed cases produces unstable annual rates, and the single-district setting limits generalisability. Finally, the absence of an independent denominator or source dataset prevented sensitivity analyses and external record validation. These biases mean that the findings should be interpreted as programme-monitoring signals, not causal estimates or precise measures of community incidence and immunity.

### Conclusion

Measles occurrence in Chereponi District showed marked but small-number-sensitive temporal variation between 2019 and 2024, with a sharp increase in suspected notifications in 2022 and the highest laboratory-confirmed incidence in 2023. These descriptive patterns occurred alongside inconsistent two-dose MCV coverage, substantial MCV1-MCV2 dropout in several years, and marked subdistrict heterogeneity; they do not establish an individual- or population-level association between coverage and measles occurrence. The district should prioritize sustained MCV2 completion through defaulter tracing, outreach, and subdistrict microplanning, alongside routine validation of administrative denominators and dose records, particularly in mobile border populations. SIA coverage should be digitised and documented alongside routine immunization indicators, and case-based surveillance should ensure complete documentation of WHO-required fields. Future studies should integrate longitudinal case-level vaccination, spatial, and SIA data to guide measles-elimination strategies in similar rural and mobile populations.

## CRediT authorship contribution statement

**Richard Dzeha:** Visualization, Validation, Software, Resources, Project administration, Methodology, Formal analysis, Data curation, Conceptualization, Writing – review & editing, Writing – original draft. **Owusu Amponsah:** Visualization, Validation, Supervision, Methodology, Conceptualization, Writing – review & editing.

## Ethics approval

Ethical approval was obtained from the Committee on Human Research, Publications and Ethics, Kwame Nkrumah University of Science and Technology, Kumasi, Ghana (CHRPE/AP/640/25).

## Funding

This research received no specific grant from funding agencies in the public, commercial, or not-for-profit sectors.

## Declaration of competing interest

The authors declare that they have no known competing financial interests or personal relationships that could have appeared to influence the work reported in this paper.

## Data Availability

The datasets used for the analysis in the current study are available from the corresponding author upon request.
